# Characterizing Antimicrobial Resistance in Chicken Pathogens: A Step towards Improved Antimicrobial Stewardship in Poultry Production in Vietnam

**DOI:** 10.3390/antibiotics9080499

**Published:** 2020-08-10

**Authors:** Nguyen Thi Phuong Yen, Nguyen Thi Nhung, Nguyen Thi Bich Van, Nguyen Van Cuong, Bach Tuan Kiet, Doan Hoang Phu, Vo Be Hien, James Campbell, Niwat Chansiripornchai, Guy E. Thwaites, Juan J. Carrique-Mas

**Affiliations:** 1 Oxford University Clinical Research Unit, Ho Chi Minh 700000, Vietnam; yenntp@oucru.org (N.T.P.Y.); nhungnt@oucru.org (N.T.N.); vanntb@oucru.org (N.T.B.V.); cuongnv@oucru.org (N.V.C.); phudh@oucru.org (D.H.P.); jcampbell@oucru.org (J.C.); gthwaites@oucru.org (G.E.T.); 2 Sub-Department of Animal Health and Production, Dong Thap 81000, Vietnam; bachkiettydt1@gmail.com (B.T.K.); hienthuydt@gmail.com (V.B.H.); 3 Faculty of Animal Science and Veterinary Medicine, Nong Lam University, Ho Chi Minh 700000, Vietnam; 4 Centre for Tropical Medicine and Global Health, Oxford University, Oxford OX3 7FZ, UK; 5 Avian Health Research Unit, Chulalongkorn University, Bangkok 10330, Thailand; Niwat.C@chula.ac.th

**Keywords:** antimicrobial resistance, minimal inhibitory concentration, chicken pathogens, bacteria, diseases, Vietnam, low- and middle-income countries

## Abstract

In the Mekong Delta of Vietnam, farmers use large quantities of antimicrobials to raise small-scale chicken flocks, often including active ingredients regarded of “critical importance’” by the World Health Organization. Due to limitations in laboratory capacity, the choice of antimicrobials normally does not follow any empirical criteria of effectiveness. The aim of this study was to highlight non-critically important antimicrobials against which chicken pathogens are likely to be susceptible as a basis for treatment guidelines. Microtiter broth dilution method was performed to determine the minimal inhibitory concentration (MIC) of 12 commonly used antimicrobials for 58 isolates, including *Ornithobacterium rhinotracheale* (ORT) (n = 22), *Gallibacterium anatis* (n = 19), and *Avibacterium endocarditidis* (n = 17). Unfortunately, internationally accepted breakpoints for resistance in these organisms do not exist. We drew tentative epidemiological cut-offs (TECOFFs) for those antimicrobial-pathogen combinations where MIC distributions suggested the presence of a distinct non-wild-type population. Based on the observed results, doxycycline would be the drug of choice for *A.* *endocarditidis* (11.8% presumptive non-wild type) and *G. anatis* infections (5.3% presumptive non-wild type). A total of 13.6% ORT isolates were non-wild type with regards to oxytetracycline, making it the drug of choice against this pathogen. This study illustrates the challenges in interpreting susceptibility testing results and the need to establish internationally accepted breakpoints for veterinary pathogens.

## 1. Introduction

Antimicrobial resistance (AMR) is a major worldwide health emergency [1]. Much of the concern derives from its impact on human health. It has been estimated that AMR-related infections will reach 10 million cases per year in 2050 [2]. There is a scientific consensus that excessive antimicrobial use (AMU) and AMR in animal populations are contributing factors to global AMR [3]. The issue of AMR in animal pathogens has received much less attention than AMR in human pathogens, and thus there is a deficit of published surveillance and research data. This is partially due to limited veterinary diagnostic capacity, especially in low and middle-income countries (LMICs) [4]. The presence of AMR traits in animal pathogens is likely to entail considerable, but yet to be quantified, economic losses derived from the failure to treat diseases [5]. Globally, over 110,000 tons of chicken meat are produced each year, making it the second most consumed type of meat in the world. Furthermore, by 2025, chicken meat production is expected to surpass that of pork [6]. A large number of bacterial pathogens can infect chicken flocks, and many such organisms are resistant to commonly used antimicrobials in farms [7]. High levels of disease and mortality are regarded as major drivers of AMU in flocks in the region, and respiratory diseases are among the most prevalent ones [8]. A number of bacterial pathogens, including colisepticaemic *E. coli*, *Avibacterium paragallinarum*, *Ornitobacterium rhinotracheale* (ORT) and *Mycoplasma gallisepticum* were detected in diseased chicken flocks in the Mekong Delta of Vietnam [9]. Previous reports have indicated extremely high levels of AMU in small-scale chicken flocks in the same region, as well as high levels of antimicrobial resistance in commensal *E. coli* of chicken origin [10,11,12]. However, there are no published data regarding levels of phenotypic resistance in chicken pathogens in flocks in the country. Current scientific consensus indicates that antimicrobials regarded by the World Health Organization (WHO) to be of critical importance for human medicine should be restricted in veterinary medicine [13] and this has recently become integrated in the policy of several countries [14,15]. Using microtiter broth dilution, we characterized the phenotypic resistance of three global chicken bacterial pathogens in the Mekong Delta (Vietnam) to commonly used antimicrobials in the area. The data on the antimicrobial susceptibility of these organisms should form the basis of treatment guidelines that prioritize the choice of antimicrobial classes that do not include critically important antimicrobials according to the WHO [16]. However, widely accepted breakpoints for the interpretation of resistance for most poultry pathogens do not exist. In veterinary medicine, setting clinical breakpoints is challenging given the range of animal species and pathogens involved. Resistance has often been defined in terms of epidemiological cut-offs (ECOFFs). These cut-offs are drawn based on the MIC distributions that have been used to distinguish between wild-type and non-wild-type populations [17]. Based on the minimal inhibitory concentration (MIC) distributions of different antimicrobial-pathogen combinations, we proposed “tentative” epidemiological cut-offs (TECOFFs) for three different poultry pathogens common in the Mekong Delta region of Vietnam. This work is the first step aiming to characterize antimicrobial susceptibility of veterinary pathogens in Vietnam. These results should be the basis of future guidelines to veterinarians and drug shop owners in the country.

## 2. Results

**Figure 1:**
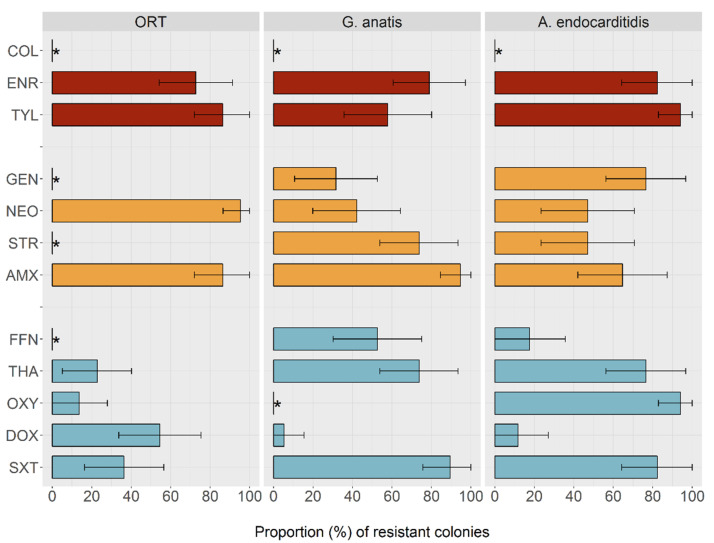
Estimated prevalence of presumptive non-wild phenotypes with regards to 12 antimicrobials among *Ornithobacterium rhinotracheale* (ORT), *G. anatis*, and *A. endocarditidis* isolates from Mekong Delta chicken flocks. Bars indicate percent of isolates that are fully resistant, with 95% binomial confidence intervals drawn around these percentages. Red = highest priority, orange = high priority, blue = highly important antimicrobial according to the WHO. Key: COL = colistin, ENR = enrofloxacin, TYL = tylosin, GEN = gentamicin, NEO = neomycin, STR = streptomycin, AMX = amoxicillin, FFN = florfenicol, THA = thiamphenicol, OXY = oxytetracycline, DOX = doxycycline, SXT = co-trimoxazole. * Tentative epidemiological cut-offs (TECOFFs) could not be established.

MIC results are shown in Supplementary Table S1 and are summarized in Table 1 and Figure 1. For 29 (80.5%) antimicrobial-pathogen combinations, we observed a bimodal (n = 18) or multimodal (n = 11) distribution. The lower mode of these suggested a wild-type sub-population, and therefore TECOFFs were proposed. For ORT, TECOFFs could be drawn for 8/12 antimicrobials tested. For four of those antimicrobials (enrofloxacin, tylosin, amoxicillin, doxycycline), the proposed TECOFFs agreed with the cut-off values reported previously [18,19,20].

Given the observed patterns, and in the absence of susceptibility testing of isolates from a given flock, we would suggest doxycycline as the drug of choice for *A. endocarditidis* infections (11.8% presumptive non-wild type) or *G. anatis* infection (5.3% presumptive non-wild type). For ORT oxytetracycline would be a good choice (13.6% non-wild type). As a second choice we would propose florfenicol (17.6% non-wild type) for *A. endocarditidis* and thiamphenicol (22.7% non-wild type) for ORT (Figure 1). 

## 3. Discussion

Susceptibility testing of bacterial animal pathogens aims to provide a rational basis for the choice of appropriate antimicrobial therapy. Based on this, the use of non-critically important antimicrobials should be prioritized. In our study, doxyxycline (tetracycline class) is likely to be effective against *A. endocarditidis* and *G. anatis* (11.8% and 5.3% presumptive non-wild types, respectively); thiamphenicol (amphenicol class) is likely to be effective against ORT (22.7% non-wild type), whereas florfenicol (amphenicol class) is likely to be effective against *A. endocarditidis* (17.6% non-wild type). Neither amphenicols nor tetracyclines are classified as critically important antimicrobials by the WHO [16].

For a considerable number (n = 7) of antimicrobial–pathogen combinations, we obtained a unimodal distribution that did not allow TECOFFs to be drawn; further, we observed a multimodal distribution for a relatively high number (n = 11) of combinations. Given the limited number of isolates tested and the uncertainty associated with the chosen interpretative criteria, our results need to be taken with great caution. Data from a larger set of isolates are therefore required to validate these TECOFFs. These results highlight the pressing need to establish internationally accepted interpretation guidelines. As in human medicine, ideally MIC data of antimicrobial–pathogen combinations should be shared across countries, and these should be updated periodically [17]. For colistin, a critically important antimicrobial “of the highest priority” according to WHO widely used in chicken production, interpretation guidelines are restricted to human pathogens [21]. Our data indicate a unimodal distribution for these organisms, and therefore TECOFFs could not be established. Based on the magnitude of the MICs for colistin, it is likely effective against *G. anatis* and, to a lesser extent, *A. endocarditidis*.

Most LMICs have limited capacity for isolating bacterial pathogens and performing antimicrobial susceptibility testing [4]. These deficiencies are particularly severe in veterinary medicine. In Vietnam, diagnostic investigations are seldom carried out in small-scale farming settings due to economic and logistic constraints. Faced with disease, farmers and their advisors often treat flocks with antimicrobials irrespective of the pathogen [5]. A complicating factor is the fact that for many bacterial infections, clinical signs are often non-specific, and mixed infections are common [10].

Since in Vietnam veterinary drug shops are the main points of supply and advice to farmers on AMU [22], results of phenotypic AMR testing of pathogens should be made available to drug shop owners and other animal-health advisors (i.e., commune animal health workers). The study presented here is limited in terms of bacterial species and production types. Therefore, we recommend expanding it to other bacterial pathogens in different production systems. This would require establishing a well-equipped, reference laboratory capable of performing micro-agglutination antimicrobial susceptibility testing and the archiving of isolates. Examination of a (representative) sufficient number of isolates should enable the establishment of reliable ECOFFs. Monitoring changes in MIC distributions over time of commonly used antimicrobials should allow the detection of emerging resistance phenotypes, as well as drafting AMU guidelines aiming at improving the efficacy of antimicrobials used in poultry production whilst preserving those that are critically important antimicrobials for human medicine.

## 4. Materials and Methods

### 4.1. Bacterial Isolates

A total of 58 bacterial isolates including *Ornithobacterium rhinotracheale* (ORT) (n = 22), *Gallibacterium anatis* biovar *haemolytica* (n = 19) and *Avibacterium endocarditidis* (n = 17) were investigated. ORT is an emerging respiratory pathogen [23]. *G. anatis* is an opportunistic pathogen that also causes diarrhea, peritionitis, oophoritis [24], as well as systemic infections with high mortality [25] in flocks. *A. endocarditidis* causes vascular as well as hepatic/spleen lesions [26]. All isolates were recovered from diseased chickens that were subjected to a diagnostic necropsy in different locations in Dong Thap province (Mekong Delta). All isolates were recovered at the Sub-Department of Animal Health (Dong Thap) diagnostic laboratory between September 2017 and September 2019. No two isolates came from the same flock. Isolates were recovered using blood agar and chocolate agar (Oxoid, Cheshire, Great Britain) incubated in 5% CO_2_ at 35 ± 2 °C for 20–44 h. The species identification of strains was performed using matrix-assisted laser desorption ionization time-of-flight mass spectrometry (MALDI-TOF MS) (Bruker, Germany). The diagnostic work was carried out under the umbrella of the ViParc project (www.viparc.org). The project was granted ethics approval by the Oxford Tropical Research Ethics Committee (OXTREC) (Minimal Risk) (Ref. 5121/16).

### 4.2. Antimicrobial Susceptibility Testing

We investigated the 12 most commonly used antimicrobials in chicken flocks in the area [27], including: colistin (COL), oxytetracycline (OXY), tylosin (TYL), doxycycline (DOX), gentamicin (GEN), amoxicillin (AMX), enrofloxacin (ENR), neomycin (NEO), streptomycin (STR), florfenicol (FFN), thiamphenicol (THA), and co-trimoxazole (SXT). The MIC of these antimicrobials was investigated for study pathogens by broth micro-dilution following Clinical Laboratory Standards Institute (CLSI) procedures outlined in VET01S [28] and M100 [29]. MIC experiments were carried out using cation-adjusted Mueller Hinton-II broth (MHB2, Sigma-Aldrich, St. Louis MO, USA) with 2.5% lysed horse blood (E & O Laboratories, Bonnybridge, UK) in 96-well plates (Corning, Corning, NY, USA). The test ranges for antimicrobials were shown in Table 1. The MICs of bacteria were recorded after 24 h (*G. anatis and A. endocarditidis*) or 48 h (ORT) incubation at 35 ± 2 °C. Reference strains *E. coli* ATCC 25,922 and *Enterococcus faecalis* ATCC 29,212 were used to verify the quality and accuracy of the testing procedures [30].

### 4.3. Data Analyses

For antimicrobial–pathogen combinations where the MIC followed a distribution suggestive of the existence of wild-type and non-wild type populations, we proposed a tentative epidemiological cut-off (TECOFF) [17]. For antimicrobial–ORT combinations not meeting that criteria, these TECOFFs were compared with those from published studies [18,19,20]. For each antimicrobial–pathogen combination, we calculated a prevalence of “presumptive non-wild-types” highlighting the antimicrobials not belonging to the WHO critical important classes. Analyses were carried out using R software (www.r-project.org).

## Figures and Tables

**Table 1:** Distribution of minimal inhibitory concentrations (MICs) of 12 antimicrobials commonly used for three chicken pathogens from the Mekong Delta of Vietnam.

		Mic Rang (µg)														Type of Distribution
		0.03	0.06	0.12	0.25	0.5	1	2	4	8	16	32	64	128	≥256	
***Ornithobacterium rhinotracheale*** **(N = 22)**	COL	-	-	0	0	0	0	0	0	0	0	14	86	-	-	Unimodal
	ENR	-	-	14	14	0	0	9	9	23	18	9	5	-	-	Bimodal
	TYL	-	0	14	5	18	32	9	0	18	5	0	0	0	0	Multimodal
	GEN	-	-	-	-	0	0	0	9	9	55	27	0	0	0	Unimodal
	NEO	-	-	-	-	5	0	0	18	5	36	32	5	0	0	Bimodal
	STR	-	-	-	-	0	0	0	9	32	41	18	0	0	0	Unimodal
	AMX	-	-	-	-	14	0	5	27	23	14	18	0	0	0	Multimodal
	FFN	-	-	0	32	64	5	0	0	0	0	0	0	0	0	Unimodal
	THA	-	-	-	0	9	32	23	14	0	0	0	0	23	0	Bimodal
	OXY	-	-	-	0	9	14	27	18	14	5	0	0	14	0	Bimodal
	DOX	-	-	-	0	5	18	23	18	32	5	0	0	0	0	Bimodal
	SXT	-	-	-	0	5	27	32	5	18	14	-	-	-	-	Bimodal
***Gallibacterium anatis* (N = 19)**	COL	-	-	0	0	5	79	11	0	0	0	5	0	-	-	Unimodal
	ENR	-	-	0	5	5	5	5	0	11	16	21	32	-	-	Bimodal
	TYL	-	-	-	-	0	0	0	0	0	5	32	11	16	37	Bimodal
	GEN	-	-	0	5	37	16	5	5	0	16	16	0	-	-	Bimodal
	NEO	-	-	-	-	0	32	5	0	11	11	0	11	5	26	Multimodal
	STR	-	-	-	-	0	0	11	16	0	0	0	5	21	47	Bimodal
	AMX	-	-	-	0	5	0	0	5	32	5	0	11	0	42	Multimodal
	FFN				0	47	0	0	0	0	5	26	16	5	0	Bimodal
	THA	-	-	-	0	11	16	0	0	0	0	0	0	16	58	Bimodal
	OXY	-	-	-	-	0	0	0	0	0	0	5	16	26	53	Unimodal
	DOX	-	-	-	-	0	0	0	32	42	21	0	0	0	5	Bimodal
	SXT	11	0	11	5	0	11	0	0	11	63	-	-	-	-	Multimodal
***Avibacterium endocarditidis*** **(N = 17)**	COL	-	-	0	0	18	29	41	6	6	0	0	0	-	-	Unimodal
	ENR	-		12	6	0	24	0	12	6	18	12	12	-	-	Multimodal
	TYL	-	-	-	-	0	0	6	0	12	29	6	18	12	18	Multimodal
	GEN	-	-	-	0	12	18	29	18	0	0	6	18	0	0	Bimodal
	NEO	-	-	-	-	0	0	24	24	6	12	29	6	0	0	Bimodal
	STR	-	-	-	-	0	0	0	18	29	6	0	6	6	35	Bimodal
	AMX	-	-	-	-	12	18	12	24	12	12	6	0	0	6	Multimodal
	FFN	-	-	-	-	76	6	0	0	0	18	0	0	0	0	Bimodal
	THA	-	-	-	-	6	12	6	0	0	0	0	0	6	71	Bimodal
	OXY	-	-	-	-	0	0	6	0	0	6	41	41	0	6	Multimodal
	DOX	-	-	-	0	12	0	41	35	0	6	0	6	0	0	Multimodal
	SXT	0	12	6	0	0	29	18	0	12	24	-	-	-	-	Multimodal

Key: COL = colistin, ENR = enrofloxacin, TYL = tylosin, GEN = gentamicin, NEO = neomycin, STR = streptomycin, AMX = amoxicillin, FFN = florfenicol, THA = thiamphenicol, OXY = oxytetracycline, DOX = doxycycline, SXT = co-trimoxazole. NC= Not calculated.

## Supplementary Materials

The following are available online at https://www.mdpi.com/2079-6382/9/8/499/s1, Table S1: Raw MIC data of all 58 chicken pathogens investigated.
